# Cumulative Childhood Adversity and Its Associations With Mental Health in Childhood, Adolescence, and Adulthood in Rural China

**DOI:** 10.3389/fpsyg.2021.768315

**Published:** 2021-11-03

**Authors:** Wensong Shen

**Affiliations:** Department of Sociology, The Chinese University of Hong Kong, Sha Tin, Hong Kong SAR, China

**Keywords:** cumulative childhood adversity, internalizing problem, externalizing problem, depression, self-esteem

## Abstract

Capitalizing on a 15-year longitudinal dataset of 9–12 years old children in rural China, this study adopts a life course perspective and analyzes cumulative childhood adversity and its associations with mental health problems from childhood to adulthood. Four domains of childhood life are selected to construct cumulative childhood adversity: socioeconomic hardship, family disruption, physical issue, and academic setback. Overall, cumulative childhood adversity significantly associates with children’s internalizing and externalizing problems as well as adults’ depression and self-esteem. However, cumulative childhood adversity has no significant relationship with internalizing and externalizing problems in adolescence. Furthermore, different domains of childhood adversity matter differently for mental health problems in different life stages. Physical issue and academic setback have the strongest association with internalizing and externalizing problems in childhood, while only socioeconomic hardship has a significant relationship with depression and self-esteem in adulthood. The relationship between cumulative childhood adversity and adult mental health problems is fully mediated by educational attainment. Finally, there is no gender difference in either the occurrence of cumulative childhood adversity or the association between cumulative childhood adversity and mental health problems.

## Introduction

Childhood adversity is a common challenge faced by children worldwide (McLaughlin, 2016). The prevalence of exposure to childhood adversity has been found to be 38–39% across high-, middle, and low-income countries (Kessler et al., 2010). Much research also demonstrates that adversity in childhood could have lifelong costs that impair learning, behavior, and health (Green et al., 2010; Shonkoff et al., 2012; Appleton et al., 2017; Edalati et al., 2017; Jakubowski et al., 2018; You et al., 2019). Despite the extensive amount of evidence linking childhood adversity to negative health consequences, debates and inconsistencies still remain regarding the definitions, measurement, and time-varying consequences of childhood adversity (Oh et al., 2018a), particularly when the contexts in which childhood adversity is defined vary.

Most of the existing studies of childhood adversity focus on western countries (Björkenstam et al., 2019), perhaps because of the richness of data in these countries. However, the social contexts of developing countries could be sharply different from those in western countries. Even within developing countries, there is a high degree of heterogeneity in social contexts. Therefore, whether the findings obtained from particular countries in the existing literature still hold in other countries remains unclear. This uncertainty calls for more empirical studies based on developing countries, particularly the longitudinal studies that reveal the complex relationships between cumulative childhood adversity and child development outcomes over a long time span.

In China, there have been some longitudinal studies about early exposure to adversity and subsequent developmental outcomes. For example, Chen et al. (2012) examine the reciprocal direct and indirect effects among aggression, peer relationships, and depression based on a sample of 1,162 children in Beijing from the third grade to the sixth grade (ages 9–12). They find that children’s initial aggression and peer isolation positively contribute to later depression, which suggests that early adverse social-behavioral conditions exacerbate children’s developmental problems. Similarly, relying on the life history framework, Lu and Chang (2019) analyze a longitudinal sample of 198 rural adolescents in China. They find that adverse environmental factors, such as parental separation, are positively associated with subsequent aggression and risk-taking behaviors.

Based on a 6-year longitudinal sample of 1,245 adolescents from 9 counties including China, Chang et al. (2019) investigate how environmental harshness and unpredictability (measured by unsafe neighborhood, negative life events, family chaos, and family income change) affects adolescents’ developmental outcomes. Results show that environmental harshness and unpredictability longitudinally corresponds to more externalizing problems and lower academic performance, and such results are invariant to countries. In another study of 206 Chinese adolescents in rural areas, Chang and Lu (2018) examine the associations of family risk factors (such as stress, parental absence, and exposure to mortality and morbidity) and subsequent psychosocial outcomes after 18 months. Results demonstrate that family risk factors are significantly linked to more risky behaviors as well as academic underperformance.

In the Chinese context, while such studies provide valuable insights on the associations between early adversity and later developmental outcomes, due to data constraints, such longitudinal studies are mainly focused on childhood and adolescence. It remains unclear how the influences of early adversity will vary or persist over a long time span from childhood to adulthood. Thus, more research is needed to reveal the long-term associations between childhood adversity and developmental outcomes not only in childhood and adolescence but also in adulthood.

Drawing on a 15-year longitudinal dataset of children in rural China, this study tries to fill these gaps. I will examine the cumulative adversity, its different domains, and its associations with mental health problems in childhood, adolescence, and adulthood. The following sections will first lay the theoretical background for research questions, and then describe the data and methods. After presenting the empirical results, this paper concludes with a discussion.

## Theoretical Background

### Definition and Measurement of (Cumulative) Childhood Adversity

Childhood adversity is often defined as the adverse childhood experiences or even trauma that could impair children’s health and wellbeing over a long time period (e.g., Patterson et al., 2014; Reid et al., 2017; Racine et al., 2020). Since adversity might take place in different aspects of life, researchers have developed many comprehensive measures, such as Adverse Childhood Experiences (ACEs), to capture the complexity of childhood adversity. While some have used the weighting methods to differentiate the importance of various adverse events, “weighting of events, either through regression-based techniques or by independent judges, does not typically improve correlations with outcomes” (Turner and Butler, 2003, p. 95). Also, “previous findings have suggested that the number of adverse events within a specified time period is more important than the novelty or types of events, and that adolescents are at greatest risk when simultaneously experiencing multiple adaptive challenges” (Davidson and Adams, 2013, p. 534). Therefore, cumulative childhood adversity, which is a composite score of a series of adverse experiences, has been extensively used in the existing research (e.g., Clark et al., 2010; Ford et al., 2011; Tan et al., 2017; Danielson and Sanders, 2018).

Cumulative childhood adversity covers a wide range of childhood life, which should include at least four domains. First, cumulative childhood adversity is most frequently measured by family disruption or dysfunction such as parental separation, divorce, abuse, or neglect (Schilling et al., 2008; Clark et al., 2010; Danielson and Sanders, 2018; Edalati et al., 2020). Second, it is also often measured by household low socioeconomic status such as economic hardship (Surtees and Wainwright, 2007; Benjet et al., 2009; Tan et al., 2017) and parents’ low levels of education and occupation (Wheaton et al., 1997; Yazgan et al., 2021). In addition to these two domains, another domain that could be integrated into cumulative childhood adversity is physical issue since physical health problems could cause huge or even lifelong stress on children. For example, in the existing literature, cumulative childhood adversity has incorporated such measures of children’s physical conditions as hospitalization, chronic disease, or poor health (Turner and Lloyd, 1995; Wheaton et al., 1997; Surtees and Wainwright, 2007; Davidson and Adams, 2013; Shen et al., 2017). Finally, educational adversity also contributes to cumulative childhood adversity since schooling and education comprises a critical part of childhood life. Children “spend more time in school than any other setting except their bed” (Eccles and Roeser, 2011, p. 225). Also, poor academic performance could result in tremendous mental distress among students (Chen et al., 2000; Roeser and Eccles, 2000; Davidson and Adams, 2013; Huang, 2015). For instance, research has showed that children with poor academic performance feel more pressure from parents, receive more criticism from teachers, and get less friendliness from peers (Shen, 2020). For these reasons, the domain of education deserves consideration when constructing cumulative childhood adversity.

While cumulative childhood adversity provides a convenient tool to investigate the consequences of overall adverse experiences in childhood, a single score might mask some critical, differential information between different types of adversity. Some research has shown that “categorization based on adversity type did appear to result in varying strength of association between each index and mental health outcomes” (Schilling et al., 2008, p. 1141). Therefore, in addition to cumulative childhood adversity, its different domains also need to be examined, which will reveal whether these different domains have equal or differential influences on child development.

### Health Consequences of Cumulative Childhood Adversity

Research to date has demonstrated a series of health and developmental problems associated with cumulative childhood adversity. For example, a meta-analysis of 35 studies shows that cumulative childhood adversity corresponds to delays in cognitive development, infection, and sleep disruption at age 20 (Oh et al., 2018b). Cumulative childhood adversity is also linked to mental health problems in later life periods. Such mental health problems might include anxiety, depression, eating disorders, self-harm behaviors, internalizing problems, externalizing problems, antisocial behavior, and personality disorder, among others (Turner and Butler, 2003; Schilling et al., 2008; Putnam et al., 2013; McLaughlin, 2016; Björkenstam et al., 2017, 2021; Steine et al., 2017; Hébert et al., 2018).

Undoubtedly, there has been an extensive amount of evidence showing the detrimental effects of cumulative childhood adversity on adolescents’ and adults’ mental health. In contrast, much less evidence is presented in the existing literature regarding whether such detrimental effects of childhood adversity change across different life stages and whether different domains of childhood adversity contribute equally to mental health in different life periods. More research is needed to address these issues.

### The Mediating Role of Educational Attainment and Moderating Role of Gender

In addition to mental health problems, research to date has found that cumulative childhood adversity impairs academic functioning like doing homework, staying calm, and curiosity in learning (Tan et al., 2017). Childhood adversity might also lower status attainment such as educational attainment (Haas, 2006; Shen et al., 2017). Some research has pointed out that “childhood adversity is associated with worse outcomes through lower adult socioeconomic status” (Jakubowski et al., 2018, p. 702). Since educational attainment is a critical component of adult socioeconomic status (e.g., King and Bearman, 2011) and also closely related to mental health (e.g., Esch et al., 2014; Mojtabai et al., 2015), it is necessary to investigate whether educational attainment mediates the relationship between childhood adversity and mental health problems in adulthood.

Furthermore, due to historical and cultural reasons, girls are often a particularly vulnerable group compared with boys. For instance, girls are more likely than boys to have abuse experiences (Mathews et al., 2017) and suffer depression (Kessler, 2003). Research on childhood adversity also finds that girls are more likely than boys to experience childhood adversity (Baglivio et al., 2014). Despite such gender differences in childhood adversity and mental health, whether girls and boys have the same relationship between childhood adversity and mental health over life periods remain unclear.

## Research Questions

**FIGURE 1:**
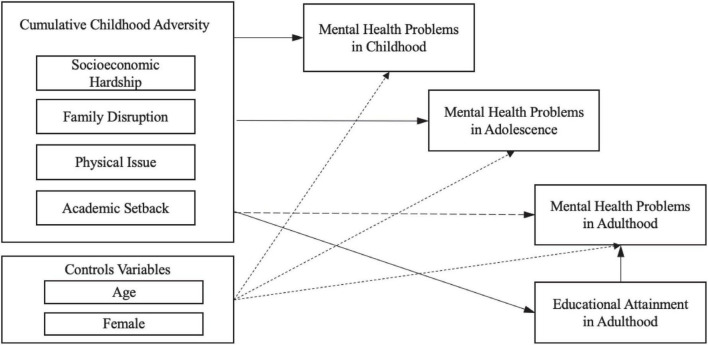
Illustration of the life-course theoretical framework.

As many previous studies (e.g., Schilling et al., 2008; Montez and Hayward, 2014), the present study adopts a life course perspective to investigate how childhood adversity relates to mental health over a long life course from childhood to adulthood. The theoretical framework is illustrated in Figure 1. Capitalizing on a 15-year longitudinal dataset that traced the development of children from childhood to early adulthood in rural China, I will investigate the following research questions:

1. What are the associations between cumulative childhood adversity and children’s mental health over a long time span including childhood, adolescence, and adulthood?
2. Do different domains of childhood adversity have similar or differential weights (or effects) on children’s short-term and long-term mental health?
3. Could educational attainment, as a critical marker of socioeconomic status, mediate the potential association between cumulative childhood adversity and mental health in adulthood?
4. Are there gender differences in the associations between cumulative childhood adversity and children’s short-term and long-term mental health?

## Materials and Methods

### Participants

The data came from the Gansu Survey of Children and Families (GSCF, 2000, 2004, 2015). GSCF is a longitudinal study of 2,000 children in 100 rural villages in Gansu. Gansu was a rural province in Northwest China where over 60% of the population resided in rural areas in 2010 (Shen et al., 2021). The percentage of rural population was even higher when the data was first collected in the year 2000. Thus, although the sample was not a representative sample of all children in China, they came from a less developed and more impoverished area and were more likely to experience adversity than the average Chinese children. Moreover, to my knowledge, this is the only available longitudinal data in China that has focused on child development an education and traced their life trajectories from childhood to adulthood in 15 years. For this reason, the data was suitable for this study.

The sampled children were first interviewed between ages 9 and 12 in the year 2000, and last interviewed in early adulthood in the year 2015. In the 2000 and 2004 waves, questionnaires were administered at schools and in homes to children, teachers, school principals, mothers, and household heads. In 2015, to reduce non-response and attrition due to out-migration, interviews were conducted in family homes during Chinese New Year, when out-migrants were likely to return home for family reunions during the festival. For respondents who did not return home during the festival, basic demographic and education information from the household head and self-reported information through phone interviews were collected. This resulted in 1,613 out of the initial 2,000 respondents successfully followed in 2015. In the 2000 and 2004 waves, both children’s internalizing and externalizing problems were measured, while in 2015, the now-adults’ depression and self-esteem were measured as the indicators of their mental health.

### Measures

In the present study, childhood adversity consisted of four domains that covered a wide range of childhood life experience: socioeconomic hardship, family disruption, physical issue, and academic setback. Each domain included three binary indicators with 1 denoting a particular adversity and 0 denoting the lack of that particular adversity. All adversity indicators were measured in the year 2000.

Socioeconomic hardship measured the socioeconomic status of a household. In extensive research, socioeconomic status comprises three indicators—education, income, and occupation (e.g., Kessler, 1982; Duncan et al., 2002; Baker, 2014; Ayoub et al., 2018). As the sample were drawn from rural areas where all children’s parents were farmers, their occupation had no variation and thus only education and income were utilized for socioeconomic hardship. Specifically, socioeconomic hardship was measured by maternal education, paternal education, and income insufficiency. Back to 2000 in the study site, the educational levels of most parents were really low. For instance, in the sample, 51% of mothers and 24% of fathers received no education at all. Thus, no education instead of a low level of education (such as primary education) was selected as the indicator of adversity. A parent who received no education was coded as 1 which indicated adversity for children. Back to the year 2000 in rural Gansu, many households still suffered from poverty and some even relied on borrowing money to afford children’s education (Shen and Hannum, 2020). Therefore, income insufficiency was another measure of socioeconomic hardship. It was obtained from the question “whether your household income in the past year was sufficient” in mother’s questionnaire. An answer of “no” was coded as 1 for income insufficiency.

Family disruption has been found to have long-term negative influences on children’s status attainment and mental health (e.g., Biblarz and Raftery, 1993; Gilman et al., 2003; Steele et al., 2009). In the existing research, family disruption is defined as the “discontinuation of cohabitation between the child’s biological parents” (Eriksen et al., 2017, p. 1080). It often includes the separation and divorce of parents (e.g., Somers et al., 2011). In GSCF, family disruption measured both the formal and informal separations of parents. Formal separation denoted the dissolution of marriage such as divorce, while informal separation denoted the separation due to migration. Therefore, family disruption included three indicators: maternal migration, paternal migration, and parental divorce. A parent was defined as being in migration if he or she was absent from home for at least 6 months in the past year (Graham and Jordan, 2011; Sun and Wang, 2016; Viet Nguyen, 2016).

Physical issue denoted the physiological problems that may directly or potentially harm children’s physical, emotional, and academic functioning. In GSCF, physical issue was measured by whether children had any chronic disease, insufficient breakfast, or myopia. Children who cannot have enough breakfast, either because of poverty or because of eating habit, tend to have low protein intake (Chitra and Reddy, 2007). “Protein malnutrition is prevalent in the developing parts of the world and children are the most affected” (Navam et al., 2014, p. 1). In China, research has shown that children who had no or little breakfast would have higher risks for malnutrition (Li et al., 2018). In GSCF, 15% of sampled children reported that they could not have breakfast enough to feel full. In this context, insufficient breakfast was taken as a marker of adversity since it was strongly related to malnutrition of children. Myopia among children and youth has become a global problem (Leo, 2017). Yet it is rarely considered as adversity because glasses to correct myopia are often easily accessible and affordable, particularly in urban areas. However, in the context of impoverished rural areas in this study, people either lacked the awareness or had insufficient financial resources to correct for children’s myopia. For instance, in this study, 20% of the sampled children reported that they had difficulties in reading blackboard or doing homework due to short-sightedness. For this reason, myopia was included as an indicator of adversity.

Academic setback denoted the problems and difficulties children encountered in their schooling and education, such as poor school performance (Davidson and Adams, 2013; Shen et al., 2017). This was measured by two questions in the data: whether children perceived their language (i.e., Chinese) performance was very poor and whether they perceived their math performance was very poor. An answer of “yes” was coded as 1 to denote a particular educational adversity. Furthermore, some children might experience grade retention. Grade retention, often due to falling behind peers, could result in a series of mental health problems such as low self-concept, misconduct in school, and depressive symptoms (Demanet and Van Houtte, 2013; Klapproth et al., 2016; Hu and Hannum, 2020). It was also adopted as an indicator of cumulative adversity in past research (e.g., Wheaton et al., 1997). Thus, grade retention was also included as a third marker of educational adversity.

These measures of (cumulative) childhood adversity, although together describing the total adverse events children encountered in particular domains of childhood life, were not necessarily correlated with each other. For instance, a child with poor math performance might not have any chronic disease, and poor math performance was also not indicative of whether parents were divorced. Therefore, Cronbach’s reliability score was not calculated for adversity measures. This is consistent with the practice in many existing studies (e.g., Schilling et al., 2008; Montez and Hayward, 2014; Björkenstam et al., 2018). In fact, the reliability of adversity measures concerns more about whether the reported adversity was truly experienced by children. Many past studies adopted retrospective data on childhood adversity and showed a good reliability (Surtees and Wainwright, 2007). Childhood adversity in GSCF were all measured in the year 2000 when children were still in childhood and when such events just took place. Thus, those adversity measures should be more reliable than the retrospective recalls.

In 2000 and 2004, children’s mental health was measured by their internalizing and externalizing behavioral problems. Internalizing and externalizing problems were measured by a set of questions adapted from the Youth-Self Report (Achenbach, 1991), edited through local piloting for cultural relevance (Liu, 2003). Each type of behavioral problems contained 18 questions, and each question was rated on a 4-point Likert scale (1–4: totally disagree, disagree somewhat, agree somewhat, and fully agree). A summative score was calculated for each type of behavioral problems, with a higher score implying more behavioral problems. For internalizing problems, the Cronbach’s alpha scores in 2000 and 2004 were 0.834 and 0.802, respectively. For externalizing problems, the Cronbach’s alpha scores in 2000 and 2004 were both 0.870. In 2000, the sampled children were in their late childhood. 4 years later in 2004, they entered their adolescence. Thus, the 2000 and 2004 measures of behavioral problems depicted their mental health from childhood to adolescence.

In 2015, the respondents’ mental health was measured by their depression and self-esteem. Depression in 2015 was measured by a shortened (10-item) form of the CES-D scale, validated and used in many studies (Andresen et al., 1994; Baron et al., 2017; González et al., 2017). For each question, a Likert scale of 1–4 denoted never, occasionally, sometimes, and often for the frequency of each depressive symptom. Thus, higher values indicated a higher likelihood or level of depression. The Cronbach’s reliability score was 0.737. Self-esteem in 2015 was measured by the widely used and validated 10-item Rosenberg Self-esteem Scale (e.g., Schmitt and Allik, 2005; Zhang and Kong, 2021). Each item was rated on a 4-point Likert scale denoting totally disagree, disagree somewhat, agree somewhat, and fully agree. A summative score was calculated and a higher score indicated a higher level of self-esteem. The Cronbach’s reliability score was 0.722.

Educational attainment in 2015 was measured by the total years of education that children had attained, transformed from a categorical variable of children’s highest level of education. According to children’s age, all sampled children should have completed formal education by 2015. Thus, the 2015 measure represented their stable or fixed educational attainment. In addition, children’s age in 2000 and gender (1 for female and 0 for male) were used as control variables. Children’s age was calculated by taking the difference between the survey year 2000 and children’s birth year reported in the household questionnaire.^1^ The descriptive statistics for all variables are listed in Table 1.

**TABLE 1:** Descriptive statistics.

	Mean/Proportion	*SD*	Min	Max	N
Cumulative childhood adversity	2.022	1.439	0	7	1,933
Socioeconomic hardship	1.145	0.959	0	3	1,971
Family disruption	0.251	0.476	0	3	1,968
Physical issue	0.253	0.480	0	3	1,990
Academic setback	0.379	0.639	0	3	1,973
Internalizing problems 2000	39.975	8.140	18	72	1,970
Externalizing problems 2000	35.295	8.877	18	72	1,976
Internalizing problems 2004	37.094	6.560	18	65	1,868
Externalizing problems 2004	31.550	7.011	18	64	1,867
Depression 2015	17.642	4.279	10	40	1,019
Self-esteem 2015	27.577	3.053	14	38	1,059
Educational attainment	11.387	3.537	0	19	1,613
Age in 2000	11.089	1.146	9	13	1,994
Female (1 = Yes, 0 = No)	0.464		0	1	2,000

*Due to age calculation formula (i.e., 2000—birth year), some children aged 12 (e.g., those born in November of 1987 and interviewed in July of 2000) could be rounded up to 13.*

### Statistical Analysis

This study utilized structural equation models (SEM) for statistical analysis. SEM has the following advantages. First, it can estimate how childhood adversity corresponds to different mental health problems simultaneously. Second, it has a convenient and powerful technique of handling missing data, i.e., the full information maximum likelihood (FIML) method. Different from the conventional multiple imputation method, the results estimated by FIML are unaffected by the imputation model and also asymptotically efficient (Allison, 2015). Third, the multiple group analysis in SEM can estimate the same model for different subgroups simultaneously, which facilitates the comparison between girls and boys for the relationships between childhood adversity and mental health outcomes. Given these advantages, SEM is a preferred method for this study.

For each life stage (e.g., childhood, adolescence, and adulthood), a model with mental health problems as endogenous variables was estimated. In each model, the error terms of the two types of mental health problems in the same life stage were also correlated to indicate that they could be both affected by some unobserved factors. Age in 2000 and gender were included as control variables (except that gender was not included as a control variable for the gender difference model). For each life stage, another model that used the four domains of childhood adversity was also estimated in order to show the differential contributions of these four domains.

Next, educational attainment was added to the adulthood model to examine whether it mediates the relationship between childhood adversity and adults’ mental health. As for gender differences, the same model was estimated for both girls and boys, and then their parameters were compared. The childhood and adolescence models used all the 2,000 observations. In 2015, only 1,613 respondents were followed, and thus the adulthood model only analyzed these 1,613 observations. FIML was used for parameter estimation and the handling of missing data. All models were saturated models and thus no goodness-of-fit test statistics were reported. To adjust for non-normality in dependent variables, robust standard errors were calculated for all models (Chou et al., 1991; Hu et al., 1992). All the SEM models were estimated by STATA 17.

## Results

Table 1 depicts the descriptive statistics. The average of cumulative childhood is 2.022, which indicates that the overall frequency of childhood adversity is not high. The most common childhood adversity is socioeconomic hardship. This is not surprising given that the sample was collected in rural areas. Academic setback is the second most frequent childhood adversity. This result illustrates the importance of incorporating educational adversity into the construct of childhood adversity when children spend so much time in school.

**FIGURE 2:**
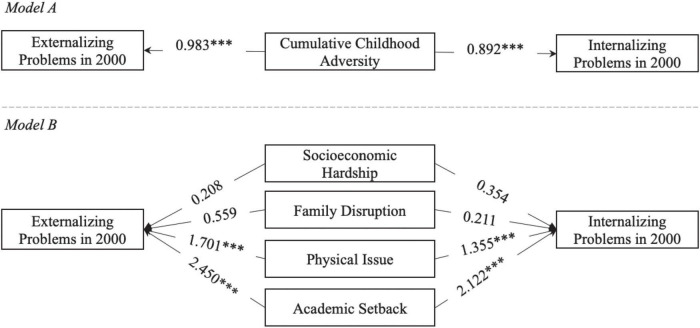
Illustration of the associations between childhood adversity and childhood mental health in childhood. Age and gender are not shown in this figure for simplicity. Numbers on arrows are unstandardized regression coefficients. * < 0.05, ** < 0.01, *** < 0.001.

Table 2 and Figure 2 show that after adjusting for children’s age and gender, cumulative childhood adversity is significantly associated with both internalizing and externalizing problems in childhood. However, when childhood adversity is decomposed to four domains, not all domains matter for children’s behavioral problems. Academic setback has the strongest association with children’s behavioral problems. The second strongest association exists between physical issue and children’s behavioral problems. In contrast, socioeconomic hardship and family disruption have no significant relationship with children’s behavioral problems in childhood.

**TABLE 2:** Maximum likelihood estimates of the associations between cumulative childhood adversity and mental health in childhood.

	Unstandardized coefficients	
	Internalizing problems in 2000	Externalizing problems in 2000
**Model A**		
Cumulative childhood adversity	0.892*** (0.123)	0.983*** (0.138)
Age	−1.050*** (0.153)	−1.454*** (0.164)
Female	−0.304 (0.358)	−0.745 (0.386)
**Model B**		
Socioeconomic hardship	0.354 (0.191)	0.208 (0.200)
Family disruption	0.211 (0.407)	0.559 (0.444)
Physical issue	1.355*** (0.374)	1.701*** (0.409)
Academic setback	2.122*** (0.266)	2.450*** (0.298)
Age	−1.092*** (0.152)	−1.507*** (0.162)
Female	−0.244 (0.357)	−0.643 (0.385)

*All independent variables were measured in 2000. Number of observations is 2,000. R-squared is 6.5% for Model A and 8.5% for Model B. Robust standard errors in parentheses.*

* < 0.05, ** < 0.01, *** < 0.001.

**FIGURE 3:**
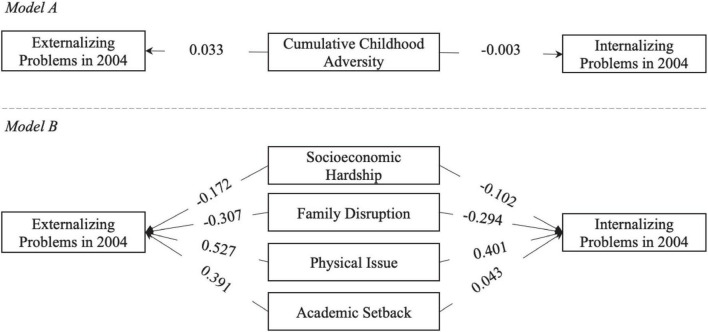
Illustration of the associations between childhood adversity and childhood mental health in adolescence. Age and gender are not shown in this figure for simplicity. Numbers on arrows are unstandardized regression coefficients. * < 0.05, ** < 0.01, *** < 0.001.

When children become adolescents, the results are listed in Table 3 and Figure 3. Surprisingly, neither cumulative childhood adversity nor any of its four domains measured in childhood has a significant relationship with behavioral problems in adolescence. One particular reason might be that when children enter adolescence and middle school, their living environments change dramatically and thus their behavioral problems also change. Such sharp changes render the previous significant relationship between childhood adversity and behavioral problems no longer significant.

**TABLE 3:** Maximum likelihood estimates of the associations between cumulative childhood adversity and mental health in adolescence.

	Unstandardized coefficients	
	Internalizing problems in 2004	Externalizing problems in 2004
**Model A**		
Cumulative childhood adversity	−0.003 (0.108)	0.033 (0.114)
Age	0.664*** (0.136)	0.333* (0.141)
Female	−0.391 (0.302)	−1.721*** (0.321)
**Model B**		
Socioeconomic hardship	−0.102 (0.165)	−0.172 (0.175)
Family disruption	−0.294 (0.308)	−0.307 (0.336)
Physical issue	0.401 (0.309)	0.527 (0.329)
Academic setback	0.043 (0.243)	0.391 (0.267)
Age	0.658*** (0.135)	0.319* (0.141)
Female	−0.384 (0.305)	−1.701*** (0.322)

*All independent variables were measured in 2000. Number of observations is 2,000. R-squared is 3.7% for Model A and 4.1% for Model B. Robust standard errors in parentheses.*

* < 0.05, ** < 0.01, *** < 0.001.

**FIGURE 4:**
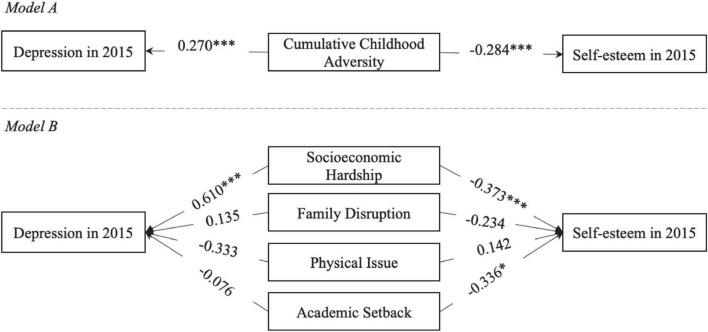
Illustration of the associations between childhood adversity and childhood mental health in adulthood. Age and gender are not shown in this figure for simplicity. Numbers on arrows are unstandardized regression coefficients. * < 0.05, ** < 0.01, *** < 0.001.

Table 4 and Figure 4 show that more cumulative childhood adversity is significantly associated with higher depression and lower self-esteem in adulthood. Yet, this significant association mainly results from socioeconomic hardship in childhood. Moreover, more academic setback in childhood significantly reduces adult self-esteem but not adult depression.

**TABLE 4:** Maximum likelihood estimates of the associations between cumulative childhood adversity and mental health in adulthood.

	Unstandardized coefficients	
	Depression in 2015	Self-esteem in 2015
**Model A**		
Cumulative childhood	0.270** (0.092)	−0.284*** (0.064)
adversity		
Age	0.219 (0.120)	−0.095 (0.086)
Female	−0.244 (0.267)	0.272 (0.190)
**Model B**		
Socioeconomic hardship	0.610*** (0.142)	−0.373*** (0.099)
Family disruption	0.135 (0.305)	−0.234 (0.212)
physical issue	−0.333 (0.263)	0.142 (0.186)
Academic setback	−0.076 (0.202)	−0.336* (0.139)
Age	0.244* (0.120)	−0.099 (0.085)
Female	−0.292 (0.266)	0.279 (0.190)

*All independent variables were measured in 2000. Number of observations is 1,613. R-squared is 2.4% for Model A and 3.8% for Model B. Robust standard errors in parentheses.*

* < 0.05, ** < 0.01, *** < 0.001.

The significant association between cumulative childhood adversity and adult mental health problems in Table 4 seems to contradict the results in Table 3. If the effect of childhood adversity on mental health has diminished in adolescence, how could the effect become significant in adulthood? Of course, one reason could be that the measures of mental health problems in adolescence and adulthood used in this study are not exactly the same. But another reason could be the mediating role of educational attainment.

**FIGURE 5:**
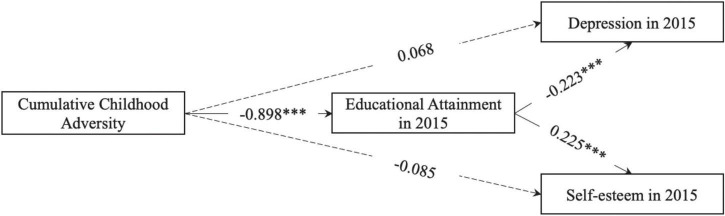
Illustration of educational attainment as a mediator of cumulative childhood adversity and mental health in adulthood. Age and gender are not shown in this figure for simplicity. Numbers on arrows are unstandardized regression coefficients. * < 0.05, ** < 0.01, *** < 0.001.

As shown in Table 5 and Figure 5, after educational attainment is included in the model, cumulative childhood adversity no longer has a direct, significant relationship with either depression or self-esteem in adulthood. Rather, cumulative childhood adversity has significant, indirect effects on adult depression and self-esteem via educational attainment. Such indirect effect sizes are comparable to the effect sizes identified in Table 4 (i.e., the adulthood model without educational attainment).

**TABLE 5:** Educational attainment as a mediator of cumulative childhood adversity and mental health in adulthood.

Parameter		Unstandardized coefficient
**Depression in 2015**		
	← Cumulative childhood adversity	0.068 (0.094)
	← Educational attainment	−0.223*** (0.041)
	← Age	0.207 (0.118)
	← Female	0.272 (0.264)
**Self-esteem in 2015**		
	← Cumulative childhood adversity	−0.085 (0.067)
	← Educational attainment	0.225*** (0.030)
	← Age	−0.085 (0.067)
	← Female	0.305 (0.184)
**Educational attainment**		
	← Cumulative childhood adversity	−0.898*** (0.056)
	← Age	−0.051 (0.074)
	← Female	−0.376* (0.165)

**Indirect effect**		

**Depression in 2015**		0.201*** (0.038)
	← Educational attainment	
	← Cumulative childhood adversity	
**Self-esteem in 2015**		−0.202*** (0.029)
	← Educational attainment	
	← Cumulative childhood adversity	

*All independent variables were measured in 2000. Number of observations is 1,613. R-squared for the model is 14.3%. Robust standard errors in parentheses.*

* < 0.05, ** < 0.01, *** < 0.001.

Finally, Table 6 describes the gender differences in the relationships between cumulative childhood adversity and mental health problems across different life stages. Consistent with previous models in each life stage, for both girls and boys, cumulative childhood adversity is significantly linked to more behavioral problems in childhood, as well as high depression and low self-esteem in adulthood, but is not significantly related to behavioral problems in adolescence. More importantly, there are no significant gender differences in the relationships between cumulative childhood adversity and mental health problems across different life stages. (The significant gender difference in the association between cumulative childhood adversity and externalizing problems in 2004 is more like a statistical artifact since the coefficients for both girls and boys are non-significant.) A further analysis shows that there is no gender difference in the occurrence of cumulative childhood adversity either (*t*-test *p*-value = 0.157).

**TABLE 6:** Gender differences in the associations between cumulative childhood adversity and mental health.

Parameter		Unstandardized coefficient		
		Female	Male	Female—Male
Internalizing problems in 2000		0.916***	0.866***	0.050
	← Cumulative childhood adversity	(0.167)	(0.178)	(0.244)
Externalizing problems in 2000		1.015***	0.950***	0.065
	← Cumulative childhood adversity	(0.189)	(0.198)	(0.273)
Internalizing problems in 2004		(0.162)	−0.156	0.325
	←Cumulative childhood adversity	0.168	(0.146)	(0.218)
Externalizing problems in 2004		0.272	−0.183	0.455*
	← Cumulative childhood adversity	(0.167)	(0.157)	(0.229)
Depression in 2015		0.296*	0.257*	0.038
	← Cumulative childhood adversity	(0.137)	(0.124)	(0.185)
Self-esteem in 2015		−0.282**	−0.284***	0.002
	← Cumulative childhood adversity	(0.101)	(0.083)	(0.131)

*All independent variables were measured in 2000. Robust standard errors in parentheses.*

* < 0.05, ** < 0.01, *** < 0.001.

## Discussion

This study focuses on cumulative childhood adversity and examines its associations with mental health problems in childhood, adolescence, and adulthood, capitalizing on a unique, 15-year longitudinal dataset in rural China. Consistent with previous literature (e.g., Turner and Butler, 2003; McLaughlin, 2016; Steine et al., 2017), cumulative childhood adversity is associated with various mental health problems in both short and long terms. However, such associations vary across different life stages and different domains of childhood adversity.

Different from a 45-year study of birth cohorts in Britain that finds unattenuated effects of childhood adversity on psychological problems over time (Clark et al., 2010), this study in China shows that cumulative childhood adversity has no significant relationship with behavioral problems in adolescence, although that relationship is significant at first in childhood. Indeed, the samples of these two studies are not comparable. But the different results regarding the time-varying effects of childhood adversity again reminded us of the importance of social contexts in which childhood adversity is studied.

Furthermore, this study shows the different values of childhood adversity as a summative score and as separate domains. As a cumulative score, it facilitates the discovery of the overall effect of childhood adversity. As separate domains, childhood adversity could reveal the unequal effects of different types of childhood adversity at different times. For example, this study finds that in childhood, physical issue and academic setback have the strongest associations with behavioral problems while in adulthood only socioeconomic hardship matters. Similarly, another study finds that family dysfunction and maltreat have different effects, with the former being more significant in early childhood and the latter being more significant in adolescence (Oh et al., 2018b). These pieces of evidence demonstrate that we cannot simply understand childhood adversity as a homogenous set of adverse experiences even if we continuously use cumulative childhood adversity.

This study also demonstrates that the effects of childhood adversity might be complex and implicit. In the long term, even if childhood adversity has no direct effect on adult mental health, it could indirectly affect adult mental health through educational attainment. This result also illuminates the importance of including education in the examination of childhood adversity. Previous research has found that among different types of childhood adversity, dropping out or failing out of school has one of the strongest effect on adult’s health (Kuhlman et al., 2018) and low school grades further heighten the risk for mental disorders among those who have experienced childhood adversity (Björkenstam et al., 2017). Similar findings are also observed in this present study. For example, academic setback has a strong association with not only children’s behavioral problems but also adults’ self-esteem 15 years later. Therefore, education—whether taken as a component of childhood adversity or a mediating or moderating role of childhood adversity’s effect—needs to be considered for the study of childhood adversity.

Some previous studies have found gender differences in childhood adversity experiences and mental health problems (e.g., Kessler, 2003; Mathews et al., 2017). However, this is not the case in the present study. In the present study, no gender difference is found in either the occurrence of childhood adversity or the association between childhood adversity and mental health problems. This further illustrates the complex relations between childhood adversity and mental health in varying contexts, and calls for more studies on the gender differences regarding both childhood adversity and its health consequences.

Of course, the differences between findings in the present study and those in other research might result from different samples. Admittedly, the sample analyzed in this study was drawn from rural China and might not be representative of the Chinese children. Thus, the results in the present study cannot be simply generalized to a different social setting. As pointed out in the introduction, more studies based on empirical evidence from developing countries are needed in order to better reveal the influences of childhood adversity in different contexts.

Another limitation in this study is that it has identified the associations between childhood adversity and mental health problems in different life stages, yet such associations are not causations. Thus, whether childhood adversity causes such mental health problems or their associations are just a result of some unobserved confounders remains unclear. Future research could develop better research design to identify more causal interpretations of the complex relationships between childhood adversity and mental health problems.

## Data Availability Statement

The original contributions presented in the study are included in the article/supplementary material, further inquiries can be directed to the corresponding author/s.

## Ethics Statement

The studies involving human participants were reviewed and approved by Harvard University and the University of Pennsylvania. Written informed consent to participate in this study was provided by the participants’ legal guardian/next of kin.

## Author Contributions

WS contributes to data analysis and manuscript writing.

## Conflict of Interest

The author declares that the research was conducted in the absence of any commercial or financial relationships that could be construed as a potential conflict of interest.

## Publisher’s Note

All claims expressed in this article are solely those of the authors and do not necessarily represent those of their affiliated organizations, or those of the publisher, the editors and the reviewers. Any product that may be evaluated in this article, or claim that may be made by its manufacturer, is not guaranteed or endorsed by the publisher.

## Footnotes

1 Due to this age calculation formula (i.e., 2000—birth year), some children aged 12 (e.g., those born in November of 1987 and interviewed in July of 2000) could be rounded up to 13. Also, due to some unknown errors in response (e.g., respondents might confuse the lunar year and solar year when reporting birth year) or data input, there were 6 observations whose calculated ages fell out of children’s true age range. The ages of these 6 observations were treated as missing values in this study. I also excluded these 6 observations from the sample for a robustness check. Whether the ages of 6 observations were treated as missing values or excluded from analytical sample, results are always consistent without any substantial change.
